# Fertility preservation in male patients subjected to chemotherapy; innovative approaches for further progress

**DOI:** 10.4274/tjod.84565

**Published:** 2017-12-30

**Authors:** Mohamed Shehata

**Affiliations:** 1 University of Cologne Faculty of Medicine, Department of General Medicine, Cologne, Germany

**Keywords:** Fertility preservation, sperm, testicle, cryopreservation

## Abstract

About 4% of male patients with cancer are under the age of 35 years. With the current increase in efficacy and safety of therapies, a growing number of young adults can achieve long-term survival. In male patients receiving systemic chemotherapy and or bone marrow transplantation, a permanent loss of fertility is a common adverse effect. The only possibility to preserve the patient’s fertility is to spare the gametes or gamete-forming cells from the chemotherapeutic effect. In adults, this can be achieved by the cryopreservation of spermatozoa with the subsequent application of assisted reproductive technology. Sperm cryopreservation is currently performed using slow-rate cryopreservation as a standard method, in which sperm cells are incubated with a cryoprotective medium and slowly subjected to hypothermia in liquid nitrogen (LN) vapor before they are placed in LN. Another technique called vitrification relies on the direct placement of the cells into LN, after being suspended in a vitrification medium. Many studies compared the clinical outcomes of both techniques and revealed equivalent results. This paper sheds light on some innovative approaches for further progress.

## INTRODUCTION

About 4% of male cancer patients are under the age of 35 years ^(1)^. With the current increase in efficacy and safety of the therapies, a growing number of young adults can achieve long-term survival^(2)^. In male patients receiving systemic chemotherapy and or bone marrow transplantation, a permanent loss of fertility is a common adverse effect. The only possibility to preserve the patient’s fertility is to spare the gametes or gamete-forming cells from the chemotherapeutic effect. In adults, this can be achieved by the cryopreservation of spermatozoa with the subsequent application of assisted reproductive technology^(3)^.

Sperm cryopreservation is currently performed using slow-rate cryopreservation as a standard method, in which sperm cells are incubated with a cryoprotective medium and slowly subjected to hypothermia in liquid nitrogen (LN) vapor before they are placed in LN. Another technique called vitrification relies on the direct placement of the cells into LN, after being suspended in a vitrification medium. Many studies compared the clinical outcomes of both techniques and revealed equivalent results^(3,4)^.

### Innovative approaches for further progress

### 1) The role of molecular biology in the sperm cryopreservation

Although most studies that evaluated the cryopreservation of sperm relied on clinical outcomes and the application of cellular biology techniques, current advances in research have revealed much about the molecular biology of the sperm and its role in sperm physiology and fertile ability. Key proteins that can directly affect sperm physiologic parameters have been identified^(5)^.

Prohibitin (PHB) is a 30-kilodalton (kDa) protein that consists of two highly homologous subunits, PHB1 and PHB2, which assemble into a ring-like structure in the mitochondrial inner membrane. The absence of PHB in somatic cells was found to be associated with mitochondrial membrane depolarization and increased generation of reactive oxygen species (ROS). Significant positive correlations were found among PHB expression, mitochondrial membrane potential, and sperm motility in normozoospermia, asthenozoospermia, and oligoasthenozoospermia samples^(6)^. Together, these observations suggest that PHB expression can be an indicator of sperm quality, and that PHB is important for sperm motility and sperm mitochondrial function.

The focal adhesion kinase protein family appears to have a direct role in protein tyrosine phosphorylation of spermatozoa, which may occur via two pathways, the canonical protein-kinase A pathway and a calcium-stimulated pathway. This protein tyrosine phosphorylation activity is a very important step in the sperm capacitation process, which is required to render the sperm competent to fertilize an oocyte^(7)^. Accordingly, further progress in the clinical practice of sperm cryopreservation could be achieved through the application of molecular biology techniques in future studies to determine the cryopreservation technique of choice.

Although short-term clinical and cellular biology studies revealed no significant differences between slow cryopreservation and vitrification, the effect on the sperm proteome might have another potential. The Zilli et al.^(8)^ research group used two-dimensional polyacrylamide gel electrophoresis and matrix-associated laser desorption/ionization time-of-flight mass spectrometry to verify whether the protein expression of sea bass sperm was affected by cryopreservation. They stated that the protein profiles differed between fresh and frozen/thawed spermatozoa, as revealed using visual inspection and image analysis software. The group identified 163 spots in fresh sperm; among them, 13 were significantly decreased and 8 were absent in cryopreserved spermatozoa^(8)^.

In addition, the generation of ROS-associated with cryopreservation could be responsible for mammalian sperm damage and the limited value of stored semen in artificial insemination^(9)^. Increased ROS generation by itself was found to affect human spermatozoa proteins in terms of expression and degradation^(10)^. A recent study revealed 27 proteins that differed significantly between control and post-thawing human spermatozoa. These proteins are thought to be involved in various sperm physiologic processes, hence, spermatozoa dysfunction after cryopreservation was suggested to be due to protein degradation and or modification^(11)^. Furthermore, the actin band in western blotting differs between fresh and post-thawing sperm,^(6)^ which might reflect its affection by the process of freezing and thawing, which ultimately affects the functionality and fertilizing ability of the sperm.

**Figure 1:**
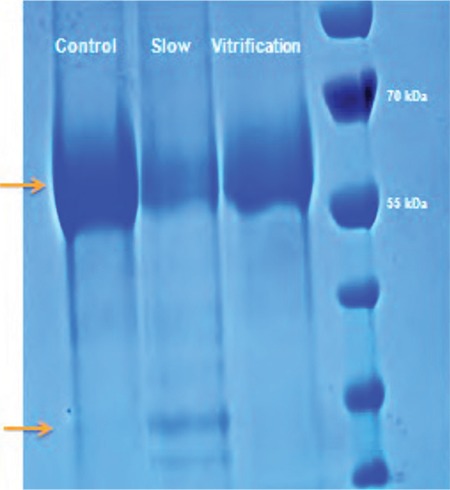
Protein extraction and separation in sodium dodecyl sulfate polyacrylamide gel electrophoresis-page showed different band patterns between control non-frozen, slow, and vitrification post-thawing spermatozoa. The most obvious band in all specimens was a protein band between 55-70 kilodalton. This band is denser in the controls than in vitrification, and denser in vitrification than in slow post-thawing spermatozoa protein extracts. The differences were visible by inspection as well as statistically significant after analysis with image-lab analyzer software (p<0.05) (Author’s own work) kDa: Kilodalton

Nevertheless, the cryopreservation of swim-up-prepared human spermatozoa with conventional slow freezing and permeating-cryoprotectants-free vitrification showed different degrees of sperm protein affection (Figure 1).

**Figure 2:**
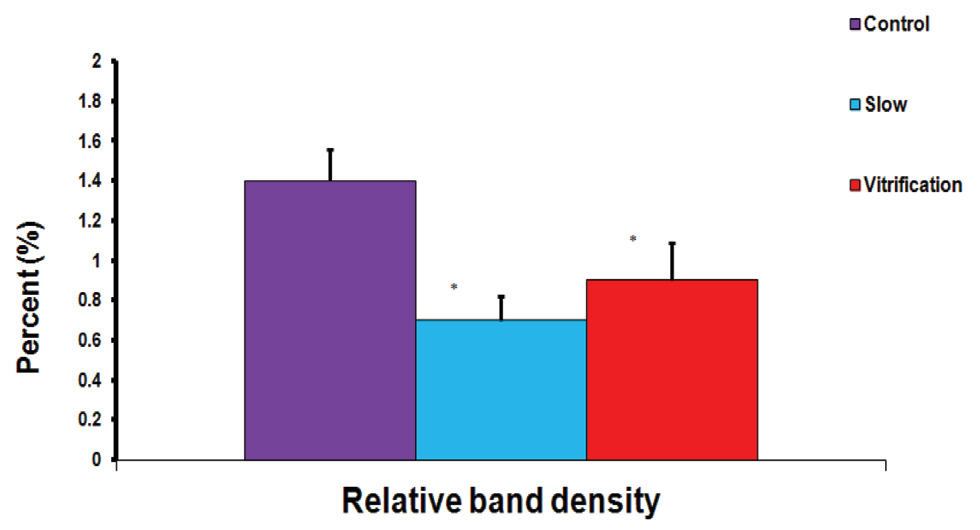
Relative band densities before and after cryopreservation. For the protein band detected in Figure 1, the relative density was decreased from ±1.4 in the controls to ±0.7 in slow freezing (50% reduction, with a significant difference p=0.003), and to ±0.9 in vitrification (36% reduction, with significant difference p=0.009). A significant difference was also found between both cryopreservation techniques (p=0.042). Asterisks indicate significant differences between marked columns with each other as well as with the control (Author’s own work)

Although no individual proteins were assessed, the application of such a simple molecular biology technique, sSDS-Page (sodium dodecyl sulfate polyacrylamide gel electrophoresis), was able to show significant differences between fresh and post-thawing spermatozoa, as well as between both cryopreservation techniques, regarding the isolated sperm proteins (Figures 1, 2). Of course, further application of mass spectrometry and proteomics analysis and or western blotting would provide more precise data about the individual affected proteins and the roles they play in controlling the physiologic parameters and fertilizing ability of sperm. However, such a level of basic and simple investigation was still able to provide reliable evidence that vitrification is superior to conventional slow cryopreservation regarding the degree of affection of sperm proteins, where conventional slow freezing was associated with more significant sperm protein degradation (Figures 1, 2).

Although further progress in this regard is expected soon, sperm cryopreservation can only help post pubertal cancer patients, who are able to provide sperm in one way or another. However, for children exposed to systemic chemotherapy, the cryopreservation of testicular tissue is the only hope for fertility preservation. Established successful cryopreservation of testicular tissue and testicular cell suspensions has been reported with either reimplantation, allowing in vivo re-establishment of spermatogenesis, or in vitro culture for ex vivo spermatogenesis.

### 2) Ex vivo testicle perfusion and cryopreservation

Though fertility restoration through testicular tissue cryopreservation has been tested and succeeded in animal models as well as in humans, little is known about the safety aspects and quality of the offspring generations^(4)^. Here, a new strategy for testicular cryopreservation is described, whose expected advantage over the currently applied techniques of testicular tissue or sperm cryopreservation would be the potential for unrestricted preservation of fertility, dual preservation of fertility and endocrine testicular functions (Table 1),^(12)^ and the preservation of the physiologic route of fertilization, i.e. sexual intercourse, without the application of assisted reproduction technology, which would at least have a significant psychological impact. Moreover, the system allows ex vivo testicle perfusion, where high doses of supportive elements and or medications could be supplemented to correct and or improve testicular functions.

The introduced procedure starts with the surgical retrieval of the testicle, where vascular catheterization and immediate perfusion begins. The used perfusates can vary according to the protocol used, for instance, minimal essential medium supplemented with human serum albumen or human tubal fluid medium supplemented with serum substitute supplement (SSS). Further supplementations could be considered according to the protocol used, e.g., ascorbic acid, antioxidants, hormones, growth factors, antibiotics, and heparin.

Immediate and continuous perfusion minimizes the risk of microthrombi formation; however, thrombolytic medications could be supplemented to the perfusates to dissolve any formed microthrombi. At this stage, the testicle has not manifested significant ischemia or oxygen or nutrient deprivation, and the vascular bed is clearly accessible.

**Figure 3:**
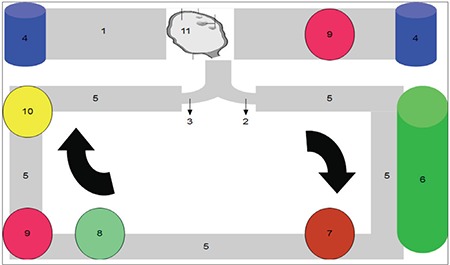
(Author’s own innovation) Diagrammatic representation of the ex vivo testicle perfusion system (author’s invention): 1. A box to enclose the testicle that works as a sealed cryovial 2. Testicular vein stump connected to the circuit using a special cannula or catheter (perfusion output) 3. Testicular artery stump connected to the circuit using a special cannula or catheter (perfusion input) 4. Reservoirs for filling the testicle-containing box with medium, the cryoprotective, vitrification, and warming solutions during cryopreservation 5. The perfusion circuit 6. Perfusate reservoir 7. Centrifugal pump (pulsatile or continuous flow) 8. Set of leukocytic and cytokines filters 9. Temperature adjustor 10. A gas exchanger to remove carbon dioxide and provide oxygen to maintain these gases in the perfusate at physiologic levels 11. The testicle subjected to perfusion and cryopreservation

Following a short period of ex vivo perfusion, the cryopreservation solution (either for slow freezing or for vitrification) can be introduced into the circuit to simultaneously fill the cleaned vascular bed of the testicle and the plastic box around the graft. This ability, together with the presence of temperature adjustors, allow the application of the cryopreservation or vitrification protocol of interest, where at the end, the graft can be stored in LN. After the patient’s survival, warming protocols could be similarly applied, where the graft can be further perfused till surgical transplantation, minimizing the ischemic reperfusion injury and providing a chance for ex vivo testicle reconditioning, using concentrated growth factors and or hormone therapies to omit cryo-injuries before re-implantation (Figure 3).

### Materials and methods of author’s experimental data

After ethical approval (University of Cologne Nr. 01-106) and patient consents, three semen samples were collected according to the recommendations of the World Health Organization (WHO) from male subjects aged between 25 and 40 years. The samples were collected by masturbation after at least 48 hours of sexual abstinence.

Semen analysis was performed according to the published guidelines of the WHO^(13)^. Samples were classified according to the following lower reference limits: 15 million spermatozoa/mL, 32% progressive motility, and a minimum of 4% morphologically normal spermatozoa.

Each semen sample was diluted 1:2 with pre-warmed (37 °C) Quinn’s Sperm Wash Medium (Sage Media, Trumbull, CT, USA) and transferred into a conical centrifuge tube (Becton Dickinson, NJ, USA) and centrifuged at 300 g for 10 minutes. The supernatant was carefully removed and discarded. The sperm pellet was resuspended in 1 mL of the same medium by gentle pipetting, followed by centrifugation again for 10 minutes at 300 g. After removing and discarding the supernatant, 1 mL of pre-warmed (37 °C) human tubal fluid medium +1% SSS was gently placed over the pellet, without disturbing it, followed by incubation for 60 minutes, at 37 °C in a 6% CO2 atmosphere, in the oblique position (45 °C). After incubation, the tube was handled gently and returned to the up-right position and the uppermost 500 µL medium, where the highly motile sperms are present, was removed into a sterile Eppendorf tube^(13)^.

Each swim-up preparation was divided into three equal parts; one was the fresh control, one for subsequent conventional slow cvitrification were performed according to the guidelines^(13)^.

### Protein extraction and SDS polyacrylamide gel electrophoresis

Fresh, slow cryopreserved and vitrified spermatozoa of the same sample and concentration were centrifuged at 300 g for 10 minutes. The supernatant parts were removed and discarded, while the cellular pellets were resuspended in 100 µL radioimmunoprecipitation assay lysis buffer supplemented with a 10% animal component-free protease inhibitor cocktail (Sigma, Munich, Germany), with vigorous shaking, vortex and sonication, when needed, to disrupt the pellet.

The protein concentration and protein amount in each sample were determined using the Bradford method. A standard curve was obtained using blank water and serial protein concentrations of 50-1600 µg/mL of bovine serum albumin. After duplicates of serial dilutions of each sample (6 µL) were equilibrated with color reagent (100 µL) (Bio-Rad Laboratories GmbH, München, Germany) for 10 minutes at room temperature, the absorbance measurements were made using a double-beam ultraviolet-visible spectrophotometer. The standard curve was plotted and the protein concentration in each sample was determined relative to the standard curve.

Equal amounts of protein were subjected to SDS-Page together with 10 µL of pre-stained page ruler protein marker (10-170 kDa) (Thermo Fisher Scientific, Bonn, Germany). The separated protein bands were stained in the gel using Coomassie blue (Thermo Fisher Scientific, Bonn, Germany). The stained gels were then scanned and the densities of the bands were determined using image-lab analyzer software (Life Science Research, Bio-Rad, München, Germany).

The relative band densities were calculated by dividing the actual band density (obtained by the image-lab analyzer) by the average of the three bands of each sample (control and post-thawing bands). The results were detectable visually, as well as with statistical calculations. For statistical analysis, an Excel data sheet (Microsoft Office 2007) for the calculation of mean and standard deviation was used. A comparison between the three treatments was performed using the Prism 6 Demo program for the determination of significant differences using the non-paired t-test, where p values less than 0.05 were considered significant.

## Figures and Tables

**Table 1:**
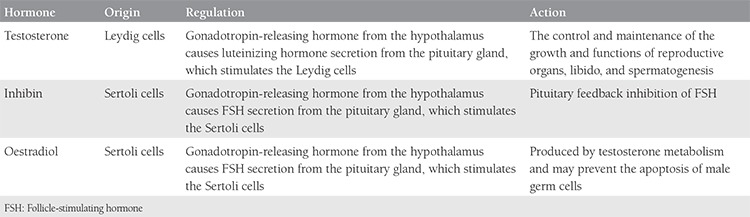
Hormones produced by the testicles^(12)^
